# Decision-making regarding total knee replacement surgery: A qualitative meta-synthesis

**DOI:** 10.1186/1472-6963-7-52

**Published:** 2007-04-10

**Authors:** Tracey O'Neill, Clare Jinks, Bie Nio Ong

**Affiliations:** 1 Primary Care Musculoskeletal Research Centre, Primary Care Sciences, Keele University, Keele, Staffordshire, ST5 5BG, UK

## Abstract

**Background:**

Knee osteoarthritis is a highly prevalent condition that can result in disability and reduced quality of life. The evidence suggests that total knee replacement surgery (TKR) is an effective intervention for patients with severe knee problems, but there is also an unmet need for this treatment in the UK. To help understand the reason for this unmet need, the aim of this study was to explore the factors that influence the decision-making process of TKR surgery by synthesising the available evidence from qualitative research on this topic.

**Methods:**

A meta-synthesis was undertaken. This involved sevens steps: getting started, deciding what is relevant to the initial interest, reading the studies, determining how the studies are related, translating the studies into one another, synthesising translations, and finally, expressing the synthesis. Second-order and third-order interpretations regarding decision-making in TKR surgery were drawn from the literature.

**Results:**

Ten qualitative studies were found and are included in the synthesis. The evidence suggests that social and cultural categories of aging have shaped the expectation of knee osteoarthritis, and this in turn shapes patients' expectations of treatment options. The role of the health care professional was the strongest theme to emerge across all ten studies. Coping strategies and life context determine short and longer-term outcomes of TKR.

**Conclusion:**

The decision-making process regarding TKR surgery is extremely complex, as patients have weigh up numerous considerations before they can make a decision about surgery. By synthesising ten qualitative studies, we have illuminated the importance of the health care professional during this process.

## Background

Knee osteoarthritis is the most common type of osteoarthritis and is a major cause of disability and reduced quality of life [1,2]. Total knee replacement (TKR) surgery is reported to be an effective intervention for people who have severe knee problems [3,4] and the number of TKR's performed in the UK has risen by over 20,000 between the years 2002 and 2004 [3]. Despite this rise, there are also reports of poor uptake of this treatment in the UK [5,6]. In addition, a number of qualitative studies [6-15] have focused on the patient experience of TKR. The findings from these studies may help to shed light on the reasons for unmet need for TKR.

The term qualitative meta-synthesis refers to "the synthesis of findings across multiple qualitative reports to create a new interpretation" [16] Finfgeld [16] refers to this as an umbrella term that includes: theory building, meta-study, grounded formal theory, theory explanation, and descriptive study. The aim of a meta-synthesis is to produce new and integrative interpretation of findings that is more substantive than those resulting from individual investigations. Qualitative researchers argue that this is required in order to make qualitative research accessible and usable in the real world of practice and policy making [17].

In quantitative studies systematic review methodology is well established, and the procedures are well developed and described. For a qualitative meta-synthesis however, there is no one standard approach. Although the Cochrane Qualitative Methods Group [18] are in discussions about the development of guidelines for the systematic review of qualitative evidence, at the moment there is no emerging consensus. According to Sandelowski and Barroso [19] the approach that you will use will depend on the purpose of your project. Some qualitative researchers argue that the methods used for quantitative systematic reviews cannot be transferred to qualitative meta-syntheses for a number of epistemological reasons [17,20,21]. Each qualitative study is unique in its own way, as it explores different complexities and contradictions within the data. Because of this uniqueness, qualitative data cannot be summarised in the same way as quantitative data. However, during the last decade there has been a large increase in the amount of qualitative studies in health sciences research, but some have argued that these studies have had little impact on health policy and practice [17]. Others claim that any efforts to summarise and synthesise the findings from individual qualitative studies could be beneficial for policy makers and managers [22]. Our aim, by undertaking a qualitative meta-synthesis, was to summarise the evidence on decision-making regarding TKR surgery, so that the findings from these studies can be more accessible to clinicians, researchers, policy makers and managers.

Models for patient decision-making extend from the paternalistic, to the shared and the informed model [23]. The paternalistic model has been the dominant approach to decision-making in the past, and applies to situations when the patient passively agrees to the doctors choice of treatment [24]. The informed model is when the doctor communicates information about all of the treatment options available, and the patients make the final decision by themself. However, research has shown that even though patients want information about their illness [23,25], they do not necessarily want to be responsible for making the final decision [26,27]. Therefore the shared model of decision-making has been advocated as the ideal, as this is when the patient and doctor work together to choose the best treatment option that is available [28].

## Methods

A number of different approaches to synthesising qualitative studies have been discussed in the literature [16,17,20,29-32]. We decided to adopt the framework used by Britten et al. [20] as their approach generates a line of argument that can be used to understand how patients make decisions about their health care use. The question this synthesis aimed to address was: 'what factors influence the decision making process of TKR surgery?

The method used by Britten et al. [20] is derived from Noblit and Hare's [31] meta- ethnography. The process is summarised below:

### Getting started

This involved an extensive systematic literature search using databases such as: Web of Knowledge, EBSCO, Cinahl, Medline, PsycINFO, Embase, Cochrane library, Ageinfo and Ageline. The key words used were: osteoarthritis, health care utilisation, beliefs, perceptions, older people, lower limb pain, motivation, qualitative research, knee pain, TKR surgery, knee replacement, prevention. The timeframe covered by the databases used in the search is 1975 to 2006. The search itself was performed between November 2005 and March 2006. A hand search of four key journals was also undertaken: Disability and Rehabilitation, Social Science and Medicine, Age and Aging, and Gerontology. The table of contents of each journal issue from 2000–2006 was reviewed.

### Deciding what is relevant to the initial interest

The lead researcher (TO) carried out the systematic literature review. During this process, TO was responsible for reviewing titles and abstracts, in order to identify papers that were relevant to the specific research question being asked. The results of this search were then fed back to the research team who agreed on the final studies that should be included in the synthesis. In line with the process used by Britten et al. [20] our synthesis assumed that these studies were of acceptable quality, and therefore no quality appraisal was undertaken.

### Reading the studies

Once the final studies were agreed, the next step was for the team to individually read each study in detail. During this process, members of the team recorded the study sample, setting, and data collection method (see table 1), in order to make comparisons.

**Table 1:** Expectations and experiences of total knee replacement in older adults

**Methods & Concepts**	Sanders et al. 2004	Toye et al. 2006	Woolhead et al. 2002	Hudak et al. 2002	Figaro et al. 2004
**Sample**	27 patients with hip/knee pain & disability.	18 participants with knee osteoarthritis listed for TKR.	25 patients on a waiting list for TKR.	17 patients who were potential for TJA (hip or knee) – but did not want the procedure.	94 black people aged 50 – 89 with knee osteoarthritis.
**Data Collection**	In-depth interviews.	Semi-structured interviews.	In-depth Interviews.	In-depth interviews.	Semi-structured interviews.
**Setting**	Survey was used to identify a sample of participants in Somerset, UK.	Patients listed for TKR at a specialist orthopedic hospital in UK.	Patients sampled from 3 orthopedic surgeon's waiting list in UK.	Participants recruited from an initial survey, conducted in Toronto, Canada.	Participants were recruited from a church or senior centre in northern Manhattan, USA.
**Concepts**					
Experience of pain	Most had experienced pain and disability for one or more decades.	Participants found it difficult to describe their pain – also reported functional loss.	Crippling and severe pain and limited mobility was identified.	_____	____
Perception of health professionals role	Did not want to bother the GP with their symptoms.	Doctor was seen as the expert.	Believed the surgeon was the expert.	Patients relied on the doctor to help them make a decision about surgery.	Doctors were seen as the gatekeepers to TKR surgery.
Expectation of treatment	Participants assumed they would not be considered as appropriate candidates for surgery because of their age.	Most participants felt that TKR surgery was the only cure.	Participants accepted that there should be a waiting list for TKR, but felt several other factors really determine who gets TKR (i.e. weight & age).	Participants believed they needed to be in constant pain and unable to move before they would consider surgery.	Most patients expressed the belief that their body should remain intact and they did not want surgery.
Expectation of condition	Participants perceived their symptoms as associated with normal aging.	Participants believed their OA would only get worse	_____	Participants had the expectation of pain with age.	OA was natural, inevitable, a sign of aging or deterioration.
Social Context & social support	______	A person's social network was an important factor in constructing the need for TKR.	_____	Participants often drew on lay sources of TJA information.	______
Comparison with others	____	____	Believed that there are people worse off than them and they should have priority for surgery.	Participants believed that there are people worse off than them.	Patients believed the economic status of blacks & whites was the reason black people did not have TKR.
Coping strategies	Relied on over the counter medications and exercise to relieve pain.	______	____	_____	Belief in god was important with regard to the outcome of surgery.

**Second order Interpretation**	Patients viewed their symptoms as a natural part of aging, and were reluctant to seek care or have surgery.	Patients make decisions about surgery based on their perception of symptoms, and depending on their life environment.	Patients have the perception that "there are probably people worse off" and they should have priority for surgery.	"The taken for granted assumption that one needs to be in constant pain and virtually unable to move before seriously considering surgery".	Participants had negative perceptions of TKR, because of the risks they associated with surgery.

**Methods & Concepts**	Marcinowski et al. 2005	Sjoling et al. 2004	Showalter et al. 2000	Woolhead et al. 2005	Clark et al. 2004

**Sample**	9 patients who had TKR – but had been waiting for up to 2 years.	9 patients after TKR, 9 patients on waiting list for hip replacement.	5 TJA patients and their spouses after surgery.	25 patients 3 months before TKR – 10 interviewed 6 months after TKR.	17 patients who were potential for TJA (hip or knee) – but they did not want the procedure.
**Data Collection**	Un-structured interviews	Un-structured Interviews.	1 focus group – to discuss their needs prior to and following TJA surgery.	In-depth interviews.	In-depth interviews.
**Setting**	Participants were contacted with a letter after surgery in a hospital in New Zealand.	Participants were recruited at a hospital in Sweden.	Participants recruited by telephone (were already scheduled for follow up visit) in USA.	Patients sampled from 3 orthopedic surgeon's waiting list in UK.	Participants recruited from an initial survey, conducted in Toronto, Canada.
**Concepts**					
Experience of pain	Living in constant pain & hurting with everyday activities.	Pain is described as dreadful and extremely disabling.	Still felt extreme pain after surgery.	Still had continued pain and immobility after surgery	Participants viewed their pain as bad, but not "'bad enough" for surgery.
Perception of health professionals role	Had a powerful faith in health professionals.	Needed to establish a trusting relationship with the doctor.	Nurses and physicians were acknowledged as important sources of information & support.	Participants did not criticise the surgeon or the surgery.	Participants wanted more information from their health care provider.
Expectation of treatment	Some viewed surgery as the only way to carry on leading a normal life.	______	Believed they would be back to normal (walking, dancing) at 56 weeks.	Patients acknowledged that TKR was major surgery and it was natural to feel pain.	Participants were concerned about the efficacy of TKR surgery.
Expectation of condition	______	______	_____	______	Participants perceived pain as natural, expected with old age.
Social Context & social support	Participants revealed that "accepting help" was a condition necessary to maintaining independence in the long run.	Having a sense of underlying support (friends & family) helped preserve continuity.	Spousal support was important during the recovery process.	Participant's perception of outcome was related to certain situations in their life (i.e. moving from a lonely neighbourhood).	Patients drew on lay sources of information.
Comparison with others	_____	_____	_____	Felt the outcome was good when they compared themselves to others worse off than them.	Relied on accounts from others who had undergone TJA surgery
Coping strategies	Having faith and a positive attitude (stoicism) allowed participants to endure the pain.	Respondents preserve the sense of living a full life by contending with what they could do.	Participants had to make adjustments to their home (putting on socks, going to bathroom) to cope after surgery.	_____	Participants 'accommodated' the pain, by making adjustments and coping.

**Second order Interpretation**	Determination, optimism, and trust sustained participants through the entire TKJA process.	Participants "put their trust in surgery to alleviate their suffering, but find it hard to live in the uncertainty inflicted by the indeterminate waiting time".	"Preparing spouses for the role changes that occur following surgery could help patients and spouses establish realistic expectations of the recovery process".	The TKR outcome was viewed positively or negatively when viewed in relation to the participant's life context or environment.	"Symptoms and information sources were the two main factors influencing patient decision making".

### Determining how the studies are related

The next step was to determine how the studies were related. To do this, the team carefully read all of the studies, and looked for common or re-occurring themes. We then met to discuss our findings, and agreed upon the final set of concepts that had emerged from the individual studies.

### Translating the studies into one another

Once the concepts were agreed upon, the next stage was to look for second-order interpretations. When developing second-order interpretations we looked for explanations that were embedded in the studies. During this step, the lead researcher (TO) read each of the studies again, looking carefully for the main explanation that emerged from each individual paper that related to the specific research question being asked. The results were then fed back to the team (CJ, PO), who agreed on the final set of second-order interpretations.

### Synthesising translations

To develop third-order interpretations and create the line of argument, we then had to consider each concept and second-order interpretation in turn. To do this we developed a table and organised the chosen concepts in one column, and the second-order interpretations in the next column (see table 2).

**Table 2:** Decision-making regarding total knee replacement surgery

**Concepts**	**Second-order interpretations**	**Third-order interpretations**
**Experience of pain** *Different ways of describing pain*	a) Patients viewed their symptoms as a natural part of aging and were reluctant to seek care or have surgery.	
**Comparison with others** *Perceived status in comparison with others*	b) Patients make a decision about surgery based on their perception of symptoms, and depending on their life environment.	
**Expectation of condition** *Perceived cause and expectation of condition*	c) Patients have the perception that " there are probably people worse off" and they should have priority for surgery.	d) Personal interpretations of social and cultural categories of aging determine judgements about being deserving for surgery.
***Expectation of treatment*** *Perception of treatment options*	e) Participants had negative perceptions of TKR, because of the risks associated with surgery.	
**Perception of health Professional's role** *Relationship with health care professional*	f) Participants "put their trust in surgery to alleviate the suffering, but find it hard to live in the uncertainty inflicted by the indeterminate waiting time".	g) Expectations of treatments are shaped by the balance between living a life on hold, and the risks associated with surgery.
**Coping strategies** *Different ways of coping with knee OA*	h) "The taken for granted assumption that one needs to be in constant pain and virtually unable to move before seriously considering surgery".	
**Social context and social support** *Life context and social support from friends and family*	i) "Symptoms and information sources were the two main factors influencing patient decision-making".	j) The decision to have TKR is linked to the amount of pain being endured, and the way that information about TKR surgery is communicated to patients.
	k) Determination, optimism, and trust sustained participants through the entire TKJA process.	
	l) The TKR outcome was viewed positively or negatively when viewed in relation to the participant's life context or environment.	
	m) "Preparing spouses for the role changes that occur following surgery could help patients and spouses establish realistic expectations of the recovery process".	n) Coping strategies and life context determine short and longer term outcomes of TKR surgery.

### Expressing the synthesis

This final step involves 'expressing the synthesis'. According to Noblit and Hare [31] expressing the synthesis is communicating the synthesis in a form that is relevant and appropriate to the audience.

## Results

### The research studies

Our systematic literature review found ten qualitative studies that investigated the experience of total knee replacement surgery. Four of these studies were carried out in the UK, two in the USA, two in Canada, one in New Zealand, and one in Sweden. Four of the studies applied a Grounded Theory approach to analyse the data; four adopted a Content Analysis approach, one applied Interpretative Phenomenology (IP), and one Interpretative Phenomenological Analysis (IPA). All of the studies were published between the years 2000–2005.

### Themes and concepts

Seven concepts were identified from the research studies (see table 1):

*Experience of pain*, or different ways of describing pain – featured in seven of the studies.

*Perception of the health professionals' role*, or the relationship between the patient and the health professional – featured in all ten of the studies.

*Expectation of treatment*, or the perception of treatment options and outcomes – featured in eight of the studies.

*Expectation of condition*, or the perceived cause of the condition – featured in four of the studies.

*Social context and social support*, or the social environment in which people live – featured in six of the studies.

*Comparison with others*, or the perceived status when compared to others in similar situations – featured in four of the studies.

*Coping strategies*, or different ways of dealing with knee osteoarthritis, featured in five studies.

Ten second-order interpretations were identified from the research studies (see table 1). As the second-order interpretations are the main explanation or theory to emerge from the study, some are direct quotes that were extracted from the findings of the individual papers.

### Decision-making regarding TKR surgery

Four third-order interpretations were constructed. When developing third-order interpretations, one has to consider each concept and second-order interpretation in turn. Although the line of argument develops from the second-order to third-order, the third-order interpretations are conceptualised after the formulation of the concepts. The concepts, therefore, shape the conceptual context when developing the third-order interpretations. For example, the literature suggests patients perceive knee osteoarthritis to be associated with normal aging. Patients also have the belief that there are people worse off than themselves and it is they who should have priority for surgery. Taken together with concepts such as, expectation of condition, and comparison with others, our third-order interpretation conceptualise this as 'personal interpretations of social and cultural categories of aging determine judgements about being deserving for surgery'. The line of argument continues with the following second-order interpretations: patients find it difficult to live with the uncertainty caused by the indeterminate waiting time of surgery, while others had negative perceptions of surgery because of the risks they associated with surgery. Taken together with the concept, expectation of treatment, our third-order interpretation conceptualises this as 'expectations of treatments are shaped by the balance between living a life on hold, and the perceived risks associated with surgery'. The line of argument continues with the following second-order interpretations: patients believed they needed to be in constant pain and virtually unable to move before they would seriously consider surgery. For others, the decision to have surgery is influenced by their perception of symptoms, and information sources. Taken together with the concepts, experience of pain, expectation of condition, and the perception of the health professional's role, our third-order interpretation conceptualises this as 'the decision to have TKR is linked to the amount of pain being endured, and the way information about TKR surgery is communicated'. The line of argument continues to include that if patients decide to have TKR surgery, it is determination, life environment, and the role of spouses, which influences expectations and outcomes. Taken together with the concepts, social context and social support, and coping strategies, our third-order interpretation conceptualises this as 'coping strategies and life context can determine short and longer-term outcomes of TKR surgery'.

A further concept that did not feature in the second-order interpretations (i.e. it was not the main conclusion from the authors of the studies), but was evident in all ten of the studies, was the patients' perception of the health professional's role. Patients wanted to be able to trust their health care professional, and would rely on their guidance and support. Many patients perceived their GP or hospital doctor as the 'expert', and wanted their advice when faced with the decision of surgery. While others believed that their GP was the gatekeeper to TKR surgery. However, if patients made the decision to have surgery, they did not want to be critical of the surgery or the surgeon. Therefore, the doctor-patient relationship is an important concept in relation to decision-making regarding TKR surgery, and this has become explicit through the process of conducting this meta-synthesis.

## Discussion

The aim of this synthesis was to explore the factors that can influence the decision-making process regarding TKR surgery from the patients' perspective. Our findings suggest that decision-making in relation to TKR surgery is extremely complex, and patients have to weigh up numerous considerations. The literature from the ten studies suggests that knee osteoarthritis is viewed as a natural part of aging, the process of 'wear and tear'. Patients make adaptations to their life and learn to cope with the pain. Social and cultural categories of aging have shaped expectations of this condition, and this in turn shapes expectations of treatment options. For instance, many patients believed that they needed to be in constant pain and virtually unable to move before they would seriously consider surgery. While others had negative perceptions of TKR surgery, because of the risks they associated with surgery.

A qualitative study conducted by Clark et al. [15] (included in this synthesis) explored elderly patients' decision-making regarding total joint replacement. They argue that decisions about total joint replacement are highly individualised, as patients constantly shift the threshold, a term they have conceptualised as the 'the moving target'. Our data synthesis and interpretations have highlighted that the doctor-patient relationship is also important during this process, and this may well influence patients shifting thresholds. The importance attached to the doctor-patient relationship has received a great deal of interest in health services research, with a significant amount of research conducted on the topic [33-36]. For example, a study that included 3,707 interviews with general practitioners and patients in six countries, found that patients believe that only family relationships are more important than the doctor-patient relationship [36]. Barry et al. [37] carried out a study to investigate patients' agendas before consultation, to assess if these agendas are voiced, and to assess the effects of unvoiced agendas on outcomes. They found that patients' agendas are complex and multifarious and most participants did not voice all of their agendas in consultations with their GP. They argue that a positive outcome will only be achieved when patients' needs have been fully articulated in the consultation, and our synthesis confirms these findings.

It has been suggested that the use of decision-making aids may affect the decision-making process and also surgical rates [38]. O'Connor and colleagues [39] conducted a systematic review of decision aids for patients facing health treatments or screening decisions. They found that decision aids are better than usual care in improving patients knowledge, comfort, and participation in decision-making, without increasing anxiety. However, a narrative review conducted by Say et al. [40] argues that patient preferences are influenced by a number of other factors, including demographic variables, the experience of health and illness, the patient's diagnosis, and the relationships they experience with health professionals. A population-based study conducted by Juni et al. in 2003 [5] found that the poor uptake of TKR in England could be due to the negative perceptions of surgery among both patients and general practitioners. Therefore doctor-patient communication could be an important factor contributing to the unmet need for TKR. However, although the shared model of decision-making is advocated as the most ideal model [28], this may not always be easy to achieve in practice.

### Strengths and Limitations

There are a number of different approaches to synthesising qualitative studies [16,17,20,29-32]. We decided to adopt the framework used by Britten et al. [20] (originally derived from Noblit and Hare [31]) so that we could identify the factors influencing the decision-making process of TKR from the patients' perspective. One of the strengths of using this approach was that we were able to look for concepts that were relevant to all ten papers. We could then translate the studies into one another, and provide further interpretations. By creating a line of argument we were able to explore the decision-making process from the patient perspective.

A further strength was that our systematic literature review found a small, but manageable number of studies in the literature. If the subject under review was more widely researched, synthesising and translating the synthesis may be more problematic due to the quantity of data and potential contradictions in the findings. There were three researchers working on this review. The lead researcher (TO) was responsible for the systematic review, and initial coding. The results of this were then fed back to the team. We found a lot of agreement amongst our team, but this could be due to the fact that the authors are from similar disciplinary backgrounds. Whether the findings would be different with the inclusion of other disciplines is difficult to say, but as yet, no guidelines have been developed with regard to the composition of review teams.

There are a number of limitations to this study. The method that we have followed allows for only one second-order interpretation from each study. However, there may be more than one key finding from an individual study, so the researcher must prioritise which one is selected. This may be addressed by returning to the authors of the studies to verify that the second order interpretation that has been chosen is the most salient. This process has also been used to verify third order interpretations [20]. However, there are many practical and logistical problems associated with this validation process (for example locating researchers), and we therefore chose not to adopt this approach.

Secondly, we did not carry out a quality appraisal on the studies that were included. A number of quality assessment tools have been developed (for example Critical Appraisal Skills Programme [41]), but there is a significant debate on whether qualitative research can be assessed using quality criteria. A hierarchy of evidence for assessing qualitative health research has recently been developed [42]. Dalya and colleagues describe four levels of a qualitative hierarchy including single case studies, descriptive studies with minimal analysis, conceptual studies and generalisable studies. However, other qualitative researchers [17] state that qualitative studies should not be excluded for reasons of quality, as there are wide variations in conceptions of what constitutes 'good quality'. Barbour [43] argues that critical appraisal of qualitative research can be reductionist, and a checklist approach can bring its own problems, unless it is embedded in the broader understanding of the rationale and assumptions behind qualitative research. Furthermore, a study by Dixon Woods and colleagues [44] has shown inconsistent agreement between reviewers of qualitative research using three quality appraisal methods. This study reported substantial variations between reviewers, and in individual reviewers who re-assessed papers using alternative methods [44]. The authors conclude that, "researchers should exercise care in how they assess quality of evidence and how they use claims about quality". With the above in mind, we decided against excluding studies on the basis of a checklist of quality criteria.

## Conclusion

This study has confirmed that Noblit and Hare's seven-step approach [31] is a useful guide when synthesising individual qualitative studies. Our data synthesis and interpretations suggest that decision-making regarding TKR surgery is extremely complex, and patients have to weigh up numerous considerations. Our findings highlight the importance of the 'doctor-patient' relationship during the process, as this was common feature throughout all ten studies included in the review.

## Competing interests

The author(s) declare that they have no competing interests.

## Authors' contributions

TO was responsible for carrying out the systematic literature review, and this was reported back to CJ and PO.

TO, CJ, and PO participated in the construction of concepts, and second and third order interpretations as a team.

All authors contributed to the development of this manuscript

All authors read and approved the final manuscript.

## Pre-publication history

The pre-publication history for this paper can be accessed here:



## References

[B1] Dieppe P, Basler HD, Chard J, Croft P, Dixon J, Hurley M, Lohmander M, Raspe H (1999). Knee replacement surgery for osteoarthritis: effectiveness, practice variations, indications and possible determinants of utilization. Rheumatology.

[B2] Sprangers MA, De Regt EB, Andries F, Van Agt HM, Bijl RV, De Boer JB, Foets M, Hoeymans N, Jacobs A, Kempen G, Miedema H, Tijhuis M, De Haes H (2000). Which chronic conditions are associated with better or poorer quality of life?. J Clin Epidemiol.

[B3] Hawker G, Wright J, Coyte P, Paul J, Dittus R, Croxford R, Katz B, Bombardier C, Heck D, Freund D (1998). Health-related quality of life after knee replacement. J Bone Joint Surg Am.

[B4] Saleh KJ, Dykes DC, Tweedie RL, Mohamed K, Ravichandran A, Saleh RM, Gioe TJ, Heck DA (2002). Functional outcome after total knee arthroplasty revision – A meta-analysis. Journal of Athroplasty.

[B5] Juni P, Dieppe P, Donovan J, Peters T, Eachus J, Pearson N (2003). Population requirement for primary knee replacement surgery: a cross sectional study. Rheumatology.

[B6] Sanders C, Donovan JL, Dieppe PA (2004). Unmet need for joint replacement: a qualitative investigation of barriers to treatment among individuals with severe pain and disability of the hip and knee. Rheumatology.

[B7] Woolhead G, Donovan J, Dieppe P (2005). Outcomes of total knee replacement: a qualitative study. Rheumatology.

[B8] Woolhead GM, Donovan JL, Chard JA, Dieppe PA (2002). Who should have priority for a knee joint replacement?. Rheumatology.

[B9] Showalter A, Burger S, Salyer J (2000). Patients and their spouses' needs after total joint arthroplasty: A pilot study. Orthopaedic Nursing.

[B10] Sjoling RN, Agren RN, Olofsson N, Hellzen RN, Asplund RN (2005). Waiting for surgery; living a life on hold – a continuous struggle against a faceless system. Int J Nurs Stud.

[B11] Marcinkowski K, Wong VG, Dignam D (2005). Getting Back to the Future: A Grounded Theory Study of the Patient Perspective of Total Knee Joint Arthroplasty. Orthopeadic Nursing.

[B12] Figaro M, Allegrante J, Russo P (2004). Preferences for Arthritis Care Among African American's "I don't want to be cut". Health Psychology.

[B13] Hudak PL, Clark JP, Hawker GA, Coyte PC, Mahomed NN, Kreder HJ, Wright JG (2002). "You're perfect for the procedure! Why don't you want it?" Elderly arthritis patients' unwillingness to consider total joint arthroplasty surgery: a qualitative study. Med Decis Making.

[B14] Toye FM, Barlow J, Wright C, Lamb SE (2006). Personal meanings in the construction of need for total knee replacement surgery. Soc Sci Med.

[B15] Clark JP, Hudak PL, Hawker GA, Coyte PC, Mahomed NN, Kreder HJ, Wright JG (2004). The moving target: a qualitative study of elderly patients' decision-making regarding total joint replacement surgery. J Bone Joint Surg Am.

[B16] Finfgeld D (2003). Metasynthesis: The State of the Art – So Far. Qualitative Health Research.

[B17] Sandelowski M, Docherty S, Emden C (1997). Qualitative Metasynthesis: Issues and Techniques. Research in Nursing & Health.

[B18] Cochrane Qualitative Methods Group (2007). http://www.joannabriggs.edu.au/cqrmg/index.html.

[B19] Sandelowski M, Barroso J (2006). Handbook for synthesizing qualitative research.

[B20] Britten N, Campbell R, Pope C, Donovan J, Morgan M, Pill R (2002). Using meta ethnography to synthesise qualitative research: a worked example. J Health Serv Res Policy.

[B21] Barbour R, Barbour M (2002). Evaluating and synthesizing qualitative research: the need to develop a distinctive approach. Journal of Evaluation in Clinical Practice.

[B22] Mays N, Pope C, Popay J (2005). Systematically reviewing qualitative and quantitative evidence to inform management and policy-making in the health field. J Health Serv Res Policy.

[B23] Deber R (1994). Physicians in health care management: The patient-physician partnership: changing roles and desire for information. Canadian Medical Association.

[B24] Charles C, Whelan T, Gafni A (1999). What do we mean by partnership in making decisions about treatment?. BMJ.

[B25] Biley FC (1992). Some determinants that effect patient participation in decision making about nursing care. Journal of Advanced Nursing.

[B26] Degner LF, Sloan JA (1992). Decision making during serious illness: what role do patients really want to play?. Journal of Clinical Epidemiology.

[B27] Hack TF, Degner LF, Dyck DG (1994). Relationship between preferences for decisional control and illness information among women with breast cancer: A quantitative and qualitative analysis. Social Science and Medicine.

[B28] Charles C, Gafni A, Whelan T (1997). Shared Decision making in the Medical Encounter: What does it mean? (or it takes at least two to tango). Soc Sci Med.

[B29] Jensen LA, Allen MN (1996). Meta-synthesis of qualitative findings. Qualitative Health Research.

[B30] Greenhalgh T, Robert G, Macfarlane F, Bate P, Kyriakidou O, Peacock R (2005). Storylines of research in diffusion of innovation: a meta-narrative approach to systematic review. Soc Sci Med.

[B31] Noblit GW, Hare RD (1988). Meta-ethnography: Synthesizing Qualitative Studies.

[B32] Thorne S, Jensen L, Kearney M, Noblit G, Sandelowski M (2004). Reflections on Methodological Orientation and Ideological Agenda. Qualitative Health Research.

[B33] Krupat E, Bell R, Kravtiz R, Thom D, Azari R (2001). When physicians and patients think alike: patient-centred beliefs and their impact on satisfaction and trust. Family practice.

[B34] Roter D, Stewart M, Putnam SLM, Stiles W, Inui T (1997). Communication patterns of primary care physicians. JAMA.

[B35] Bensing J, Tromp F, Dulmen S, Brink-Muinen A, Verheul W, Schellevis F (2006). Shifts in doctor-patient communication between 1986 and 2002: A study of videotaped General Practice Consultations with hypertension patients. BMC Family Practice.

[B36] Magee M Relationship-Based Health Care in the United States, United Kingdom, Canada, Germany, South Africa and Japan A Comparative Study of Patient and Physician Perceptions Worldwide. Presented at the World Medical Association "Patient Safety in Care and Research".

[B37] Barry C, Bradley C, Britten N, Stevenson F, Barber N (2000). Patients unvoiced agendas in general practice consultations: qualitative study. BMJ.

[B38] Phelan E, Deyo R, Cherkin D, Weinstein J, Kreuter W, Howe J (2001). Helping patients decide about back surgery: a randomized trial of an interactive video program. Spine.

[B39] O'Connor A, Rostom A, Fiset V, Tetroe J, Entwistle V, Llewelyn-Thomas H, Holmers-Rover M, Barry M, Jones J (1999). Decision aids for patients facing health treatment or screening decisions: systematic review. BMJ.

[B40] Say R, Murtagh M, Thomson R (2006). Patients' preference for involvement in medical decision-making: A narrative review. Patient Education and Counseling.

[B41] CASP CASP Critical Appraisal Tool. http://www.phru.nhs.uk/casp/critical_appraisal_tools.htm.

[B42] Dalya J, Willis K, Small R, Green J, Welch N, Kealy M, Hughes E (2007). A hierarchy of evidence for assessing qualitative health research. Journal of Clinical Epidemiology.

[B43] Barbour R (2001). Checklists for improving rigour in qualitative research: a case of the tail wagging the dog. BMJ.

[B44] Dixon-Woods M, Sutton A, Shaw R, Miller T, Smith J, Young B, Bonas S, Booth A, Jones D (2007). Appraising qualitative research for inclusion in systematic reviews: a quantitative and qualitative comparison of three methods. Journal of Health Services Research and Policy.

